# Salol

**Published:** 1891-07

**Authors:** Alfred Eichler

**Affiliations:** San Francisco, Cal.


ARTICLE IV.

### SALOL.

BY ALFRED EICHLER, M. D., SAN FRANCISCO, CAL.

Salol is one of the recent additions to our materia medica, but its merits are now almost universally admitted. It was discovered and first used by Swiss scientists, in 1886, and since then its use has spread all over the world. It is a white, crystalline powder, obtained by the action of carbolic acid on salicylic acid, therefore being, in chemical nomenclature, a salicylate of phenol. In composition, five grains of salol are equal to about two grains of carbolic acid and three grains of salicylic acid. Therapeutically, its effects are entirely different from those of either agent administered alone, depending on the fact that its constituents are united by chemical union, and represent, therefore, a different body, Salol possesses an odor similar to oil of wintergreen. It tastes like carbolic acid; it is soluble in water only in a very small measure, therefore watery solutions are impracticable. In alcohol, ether or oils, salol is very soluble. Further, it is interesting to know that this substance, although crystalline, cannot be reduced to a very fine powder, because the crystals have a tendency to adhere. The odor of salol may be noticed, after its administration, in the excretions as well as in the expired air. The dose differs, according to circumstances, from 15 to 120 grains a day. It should be remarked, however, that a case has been reported in which a half drachm of salol, administered to a previously healthy person, and distributed in five-grain doses over the day, has produced a severe rash over the whole body, which disappeared spontaneously in five or six days (*Medical Record*, March 3, 1888). A case of poisoning has also been reported, in which a young man had swallowed at once two drachms of salol. The resulting coma was followed by death. At the autopsy, degenerative changes were noted in the kidneys, as might be expected; the harm done was attributed to the carbolic acid contained in salol (*Medical Record*, October 11, 1890.)

As regards the method of administration of salol, it is best dispensed in pills, compressed tablets, capsules or emulsion. For external use lanoline, or a mixture of equal parts of lanoline and olive oil, offers a very good menstrum; if used externally in ointment form, it is usually prescribed in the strength of one part in ten of the ointment base.

Salol may be looked upon as a very efficient antiseptic, antipyretic and anti-rheumatic. When ingested, it is insoluble in the acid gastric juice, but it is dissolved in the alkaline intestinal secretion, being perhaps split up by the alkaline elements of the bile into its original constituents, which are then absorbed and taken into the circulation, the carbolic acid being rendered non-poisonous by the change into a phenate (carbolate) of soda. This assumption is corroborated by the fact that salol is readily decomposed by weak alkalies like bi carbonate of soda—a point which should be remembered in prescribing. After absorption and diffusion through the system, the original constituents may be detected in the urine, the carbolic acid imparting the characteristic green color of phenoluria. As salol passes the stomach unchanged, experiments can be made with it in determining the activity of the stomach in propelling food. This test has been taken advantage of in dilatation of the stomach, and is available in all diseases where the motor power of the stomach is thought to be impaired. Of course, physiological tests of this kind depend so much on varying circumstances that definite conclusions can be arrived at only when a sufficient series of control experiments with healthy individuals is also carried out.

The antiseptic effects of salol are easily enough understood on considering that it is composed of two of the most powerful antiseptics now in our possession. As an antipyretic, it acts on account of being a derivative of salicylic acid, which itself has great heat-reducing virtues. Salol, however, is more easily tolerated by the stomach than salicylic acid, as it is insoluble and non-irritant; while the latter is a strong irritant of mucous membranes, and, therefore, cannot be tolerated by the stomach for any length of time in doses sufficiently large to produce good effects. That the salicylic acid is the antipyretic factor of salol is readily proved by the clinical fact that salol acts as an antipyretic only in large doses, twenty to thirty grains, containing from twelve to eighteen grains of salicylic acid. Small doses of salol reduce heat only in a very small measure. The carbolic acid and salicylic acid, into which salol is separated in the intestine, are eliminated mostly through the kidneys, as carbolate of soda and unchanged salicylic acid. Thus the urine is made antiseptic—a fact which has been taken advantage of in genito-urinary surgery.

The Therapeutic action of salol resembles, to some extent, that of salicylate of soda. In comparing the action of the two remedies, much similarity may be observed, with some points in favor of salol. They are both excellent anti-rheumatics, salol, however, acting best in subacute rheumatism, especially when a short course of salicylate of soda has preceded its use. It will lessen the fever quickly, cause the pain to disappear, and the swelling will be reduced. As already mentioned, it acts better in chronic cases than in acute. For this purpose, it should be given in doses of not less than fifteen grains every three hours, preferably in wafer or capsule. Salol is especially efficient in those forms of rheumatism called lumbago. Sciatica, that uncertain disease, a veritable touchstone for medical skill, composed so often of neuralgic, rheumatic and malarious elements, often yields to large doses of salol.

In diseases of the alimentary canal, salol is also very efficient. It acts in the stomach merely as a mechanical antiseptic, similar to subnitrate of bismuth. In the intestinate it becomes a full and strong antiseptic. This has been made use of to treat various diseases of the intestine, like dysentery and diarrhœa. It is advisable here to combine salol with other suitable agents. For chronic diarrhœa, in which a cleansing of the intestinal tract may be desirable, it is good practice to order fifteen grains of salol in an ounce of castor oil, to be given at a dose; or, when pleasanter medication is desired, one may prescribe:

| | | |
|---|---|---|
| R | Salol - - - - | ℈ii |
| | Ol. ricini - - - | f℥iss |
| | Syr. rhei - - - - | f℥ss |
| | Pulv. acaciæ - - - | ℥ss |
| | Aquæ cinnamomii - | q.s. ad f℥iv |

M. Fiat emulsio. Sig. A tablespoonful every hour, until the bowels move very freely.

In typhoid fever salol has been used, but with no more success than other remedies. A disease running a regular course like typhoid fever will not mend its ways, or be aborted, under any one mode of treatment. Salol, however, has proved quite efficient to moderate the diarrhœa, and in conjunction with other therapeutic means, like cold ablutions and wet packs, with free stimulation and proper nourishment, it becomes a valuable adjunct to treatment.

Diarrhœa infantum offers a good field for the administration of salol. It is especially useful when the intestinal canal has been severely irritated by the products of faulty digestion. It may be ordered then in small doses alternating with antacid remedies, like chalk mixture and bismuth.

Affections of the genito-urinary organs are often improved by the exhibition of salol. The most common of all, gonorrhœa, is much more tractable to treatment with salol than without it. The main good is rendered by making the urine antiseptic and unirritating by its administration. It acts thus considerably better than injections of antiseptic substances, being practically an injection from within outward. In treating a case of gonorrhœa with salol, it can be given in conjunction with the balsam of copaiba, usually prescribed. It may be ordered as in the following formula.

R Saloli - - - - ʒ vi

Bals. copaibæ - - - fʒ iss

Syr. simpl.

Mucilaginis acaciæ - aa f℥ iij

Tinct. lavandul co. - q.s ad f℥ viij

M. Sig. A tablespoonful three times a day, one hour after meals.

Large doses will prove more efficient for this purpose than small ones. The good effects are produced entirely by the action of the antiseptic and bland urine upon the inflamed urethra. Weak antiseptic injections into the urethra from without can also be used at the same time, and will assist in securing a speedy cure. It has been shown that salol, thus used, will shorten the course of the disease materially. Chronic cases improve under judicious use of this agent, if it be used with sufficient persistence. The urine appears, after the ingestion of these large doses of salol, in a much darker color, ranging from olive green to a blackish tint; that is caused by the carbolic acid contained in the urine, and need not be feared. Painful micturition is alleviated early, and the discharge is lessened promptly; in short, the acute inflammatory stage subsides much more quickly with, than without, salol.

In other diseases of the genito-urinary organs salol also deserves a careful trial. In operating for stricture of the urethra, it is advisable to have the patient take it one or two days before the operation, to render the whole urethra aseptic. Perhaps the infiltration of tissues, so feared after operations in this region, might be rendered innocuous, if salol were given beforehand.

In diseases of the bladder it also proves useful. In cystitis it leads quickly to amelioration of the symptoms, and thus proves valuable in this troublesome and painful complaint.

Salol is of great value in treating different affections of the air passages. As the characteristic odor of salol may be noticed in the expired air, it is quite right to assume that it exerts a local influence besides the constitutional impression. It is of great value in the treatment of acute catarrhal conditions, for the treatment of which it may be combined with various other suitable agents. Terpin hydrate, for instance, acts well thus combined. The two substances can be dispensed in small doses in capsules, and given alternately every two or three hours. If salol alone is given, it will often be found to be acting like a charm. It soothes irritation, stimulates free secretion, and permits the patient to expectorate freely. Patients often remark when salol is given, that the medicine raises the phlegm quickly. It is also recommended in tonsillitis, as it will shorten the duration, besides lessening the fever, quieting the attending pain and relieving painful deglutition. In this affection an emulsion would seem to be the best mode of prescribing the drug.

During the recent invasion of influenza, salol proved of great benefit to the many afflicted. It was quite often prescribed, especially in that form of the disease simulating anginas and catarrhal sore throat. Alleviation of the rheumatic symptoms also quickly followed its use. In the treatment of other infectious diseases, salol has been found useful.

As already mentioned, in typhoid fever salol will assist in procuring asepsis of the intestines. In scarlet fever, it has also been prescribed, and it is believed continued administration of this drug will aid in shortening the duration of the disease, by eliminating the infectious material, besides reducing the temperature.

Externally applied, salol gives excelleet results in some diseases of the skin. For a simple erythema, it may be used rubbed up with starch to a dusting powder. The powder form, however, is not very eligible, as salol has a tendency to ball together and form lumps. It is more suited to a paste. As it is white in color, it may be freely applied about the face. In sycosis of the bearded portion, if it is thus applied, it will cause good results. A good formula is as follows:

| | | |
|---|---|---|
| R | Saloli - - - - | ʒ ij |
| | Zinci oxidi | |
| | Pulv. amyli - - - | aa ʒ iv |
| | Lanoline - - - - | ℥ i |

Of course salol may be added to this in any strength, providing the fatty vehicle is also increased. It is entirely innocuous when thus applied, probably not being absorbed through unbroken surfaces, and acting only as an antiseptic protective. The latter quality makes it also an admirable application in burns and scalds. It is then best used by dissolving it in some fatty oil, preferably olive oil, afterwards pouring it liberally over the injured surface, which is to be covered with soft lint.

A summary of all these properties will show salol to be a very useful addition to our materia medica. It is to be regretted that it is, like most other late additions, a proprietary article, imported from Europe and one which our laws prevent us from preparing ourselves; as a consequence it costs more than it would if it could be manufactured here.—*Med. and Surg. Reporter.*

